# Alpha-ketoglutarate (AKG) lowers body weight and affects intestinal innate immunity through influencing intestinal microbiota

**DOI:** 10.18632/oncotarget.17132

**Published:** 2017-04-16

**Authors:** Shuai Chen, Peng Bin, Wenkai Ren, Wei Gao, Gang Liu, Jie Yin, Jielin Duan, Yinghui Li, Kang Yao, Ruilin Huang, Bie Tan, Yulong Yin

**Affiliations:** ^1^ Key Laboratory of Agro-Ecological Processes in Subtropical Region, Institute of Subtropical Agriculture, Chinese Academy of Sciences, Changsha, China; ^2^ National Engineering Laboratory for Pollution Control and Waste Utilization in Livestock and Poultry Production, Hunan, China; ^3^ Hunan Provincial Engineering Research Center for Healthy Livestock and Poultry Production, Hunan, China; ^4^ Scientific Observation and Experimental Station of Animal Nutrition and Feed Science in South-Central, Ministry of Agriculture, Hunan, China; ^5^ Hunan Co-Innovation Center of Animal Production Safety, Changsha, Hunan, China; ^6^ University of Chinese Academy of Sciences, Beijing, China

**Keywords:** alpha-ketoglutarate, cryptdin, intestinal microbiota, intestinal immunity, Pathology Section

## Abstract

Alpha-ketoglutarate (AKG), a precursor of glutamate and a critical intermediate in the tricarboxylic acid cycle, shows beneficial effects on intestinal function. However, the influence of AKG on the intestinal innate immune system and intestinal microbiota is unknown. This study explores the effect of oral AKG administration in drinking water (10 g/L) on intestinal innate immunity and intestinal microbiota in a mouse model. Mouse water intake, feed intake and body weight were recorded throughout the entire experiment. The ileum was collected for detecting the expression of intestinal proinflammatory cytokines and innate immune factors by Real-time Polymerase Chain Reaction. Additionally, the ileal luminal contents and feces were collected for 16S rDNA sequencing to analyze the microbial composition. The intestinal microbiota in mice was disrupted with an antibiotic cocktail. The results revealed that AKG supplementation lowered body weight, promoted ileal expression of mammalian defensins of the alpha subfamily (such as cryptdins-1, cryptdins-4, and cryptdins-5) while influencing the intestinal microbial composition (i.e., lowering the Firmicutes to Bacteroidetes ratio). In the antibiotic-treated mouse model, AKG supplementation failed to affect mouse body weight and inhibited the expression of cryptdins-1 and cryptdins-5 in the ileum. We concluded that AKG might affect body weight and intestinal innate immunity through influencing intestinal microbiota.

## INTRODUCTION

Alpha-ketoglutarate (AKG) is a keto acid synthesized by deamination of glutamate (Glu) and an important intermediate of the tricarboxylic acid cycle. It has various physiological functions, including acting as an antioxidant [1, 2] or as an anticancer agent [3, 4] and enhancing host-defense [5]. Recently, AKG has been reported to modulate intestinal energy status, amino acid metabolism, integrity and even immunity [6]. For example, AKG regulates cellular energy status through inhibiting adenosine triphosphate synthase and reducing oxygen consumption in mammalian and Caenorhabditis elegans cells [7]. AKG has also been reported to improve the energy status of the intestinal mucosa in lipopolysaccharide (LPS)-challenged pigs [8]. AKG supplementation increases protein levels, the ratio of villus height to crypt depth and the activation of the mammalian target of rapamycin pathway in intestinal mucosa, but decreases intestinal 70 kilodalton heat shock protein expression in LPS-challenged pigs [9]. Recent compelling investigations have shown that glutamine (Gln) and arginine (Arg), which can convert to AKG indirectly, regulate intestinal innate immunity by activating multiple signaling pathways and modulating the intestinal microbiota in a mouse model [10, 11]. However, the role of AKG in intestinal innate immunity and its effect on the intestinal microbiota remain unclear. Knowledge of the influence of AKG on intestinal microbiota and intestinal immunity is critical to enhancing our understanding of the crosstalk among nutrition, immunity and the microbiota. As Arg and Gln are two important regulators of AKG metabolism, we hypothesized that AKG also affects intestinal innate immunity and the intestinal microbiota. Thus, the current study explores the influence of oral AKG administration in intestinal innate immunity and intestinal microbiota in a mouse model. The results demonstrated that AKG supplementation lowers body weight and influences the variety and composition of the intestinal microbiota in addition to intestinal innate immunity, such as promoting ileal expression of mammalian defensins of the alpha subfamily.

## RESULTS

### AKG lowered body weight

**Figure 1:**
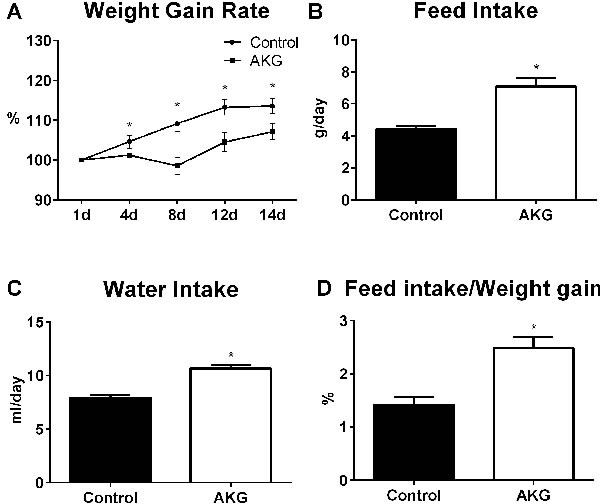
AKG supplementation lowers mouse body weight **A**. Average weight gain rate in the control group and AKG group (*n* = 30). **B**. Average feed intake in the control group and AKG group (*n* = 30). **C**. Average water intake in the control group and AKG group (*n* = 30). **D**. The ratio of feed intake to weight gain in the control group and AKG group (*n* = 30). Mice in control group received normal drinking water for 2 weeks, while mice in AKG group received AKG supplementation water (10g/L). The statistical analyzing between two groups was performed by the Student's *t*-test. *Indicates a statistically significant difference between the two groups (*P* < 0.05).

The feed intake, water intake and body weight of mice were monitored. AKG intake was 3.80±0.44 g/kg body weight per day. Compared to the control group, AKG supplementation lowered the body weight gain rate of mice (*P* < 0.05) (Figure 1A). Average feed intake, water intake, and the ratio of feed intake to weight gain in the AKG group were significantly higher than in controls (*P* < 0.05) (Figure 1B-1D).

### AKG influenced intestinal microbiota

**Figure 2:**
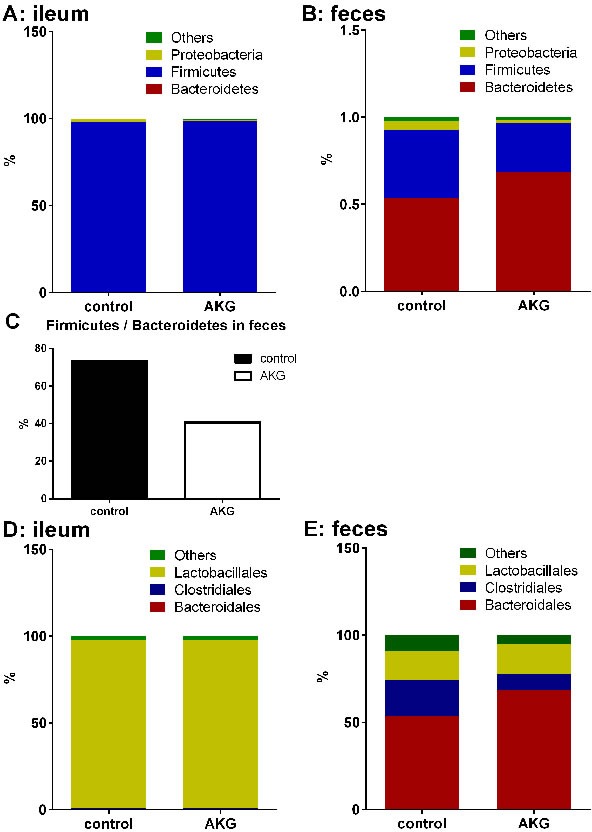
AKG supplementation influences intestinal microbiota **A**.-**B**. The microbial composition in the ileum and feces in the Control group and AKG group in the phylum (*n* = 6). **C**. The ratio of Firmicutes to Bacteriodetes in the feces of the Control group and AKG group (*n* = 6). **D**.-**E**. The microbial composition in the ileum and feces in the Control group and AKG group in the order (*n* = 6). Mice in control group received normal drinking water for 2 weeks, while mice in AKG group received AKG supplementation water (10g/L).

Intestinal microbiota cluster analysis was processed using taxon-dependent analysis to investigate the taxonomy of the intestinal microbiota of the control group and the AKG group. In both groups, six phyla were detected in the ileal microbiota, and ten phyla were found in the fecal microbiota (Supplemented file_1). Firmicutes dominated in the ileal microbiota, at 97.69% and 98.56% (Figure 2A). Bacteroidetes and Firmicutes were dominant in the fecal microbiota in both the control group (53.43% and 39.23%, respectively) and the AKG group (68.58% and 27.97%, respectively) (Figure 2B). The ratio of Firmicutes to Bacteroidetes was lower in the AKG group than in the control group, with a percentage of 32.64% in feces (Figure 2C).

For the fecal microbiota, eighteen orders were detected, including eighteen orders in the control group and eighteen in the AKG group. Eleven orders were found in the ileal microbiota, including ten orders in the controls and eight orders in the mice with AKG supplementation (Supplemented file_1). Bacteroidales, Clostridiales and Lactobacillales were the most abundant orders detected in the feces of both groups, with relative percentages of 53.42%, 20.88%, 16.74%, respectively, in the control group and 68.57%, 9.03%, 17.21%, respectively, in the AKG group. Lactobacillales was dominant in the ileal microbiota, with a relative percentage of 97.35% in the control group and 97.00% in the AKG group. In addition to Lactobacillales, Desulfovibrionales (1.46%) and Bacteroidales (0.46%) were dominant in the control group, whereas Erysipelotrichales (1.06%) and Clostridiales (0.50%) dominated in the AKG group (Figure 2D-2E).

Collectively, AKG supplementation affects the intestinal microbiota by changing the microbial composition in mice. In particular, a shift in the Firmicutes-to-Bacteroidetes ratio to favor Bacteroidetes in the feces was observed.

### AKG lowered body weight through intestinal microbiota

**Figure 3:**
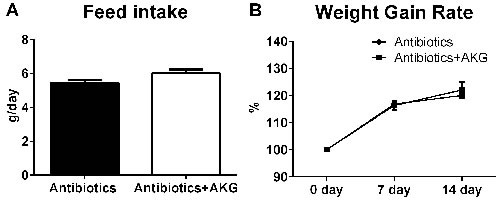
AKG supplementation fails to affect the mouse body weight in antibiotics treated mouse model **A**. Average feed intake in the antibiotics group and antibiotics+AKG group (*n* = 30). **B**. Average weight gain rate in the antibiotics group and antibiotics+AKG group (*n* = 30). Mice in antibiotics group received antibiotics-supplemented drinking water (1 g/L ampicillin; 450 mg/L streptomycin; 200 mg/L gentamicin) for 2 weeks, while mice in AKG group received water supplemented with antibiotics and AKG (10g/L AKG; 1 g/L ampicillin; 450 mg/L streptomycin; 200 mg/L gentamicin). The statistical analyzing between two groups was performed by the Student's *t*-test.

We hypothesized that AKG supplementation lowered the body weight of mice through influencing the intestinal microbiota. To verify this hypothesis, we studied the effect of AKG supplementation on body weight in antibiotic-induced ‘germ-free’ mice. In the antibiotic-treated model, the results revealed little difference in the average feed intake between mice supplemented with AKG and non-supplemented mice (*P* > 0.05) (Figure 3A). Furthermore, the body weight gain rate was similar between the two groups (*P* > 0.05) (Figure 3B). Therefore, AKG supplementation may lower mouse body weight through its effects on the intestinal microbiota.

**Figure 4:**
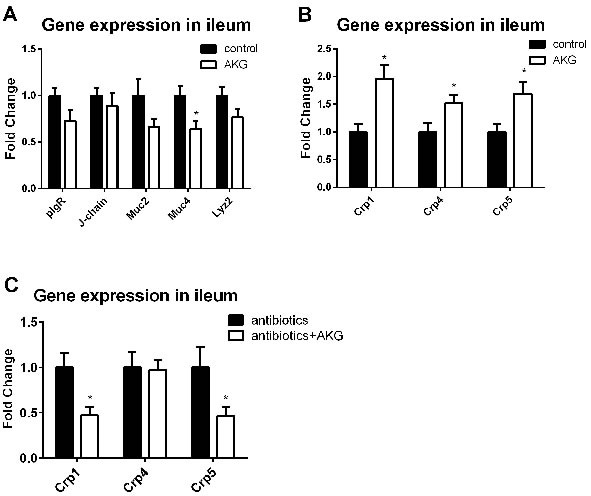
AKG supplementation affects intestinal innate immunity may through intestinal microbiota **A**. Expression of innate immune factors (Il-17, Ifn-γ, pIgR, J-chain, Muc2, Muc4, Crp1, Crp4, Crp5, and Lzy2) in the control group and AKG group (*n* = 12). Mice in control group received normal drinking water for 2 weeks, while mice in AKG group received AKG supplementation water (10g/L). **B**. Expression of α-defensins (such as Crp1, Crp4, and Crp5) in the antibiotics group and antibiotics+AKG group (*n* = 12). Mice in antibiotics group received antibiotics-supplemented drinking water (1 g/L ampicillin; 450 mg/L streptomycin; 200 mg/L gentamicin) for 2 weeks, while mice in AKG group received water supplemented with antibiotics and AKG (10g/L AKG; 1 g/L ampicillin; 450 mg/L streptomycin; 200 mg/L gentamicin). The statistical analyzing between two groups was performed by the Student's *t*-test. *Indicates a statistically significant difference between the two groups (*P* < 0.05). Abbreviations: pIgR, polymeric immunoglobulin receptor; J-chain, immunoglobulin joining chain; Muc2, mucin-2; Muc4, mucin-4; Lzy2, lysozyme 2; Crp1, cryptdins-1; Crp4, cryptdins-4; Crp5, cryptdins-5.

### AKG affected intestinal innate immunity through intestinal microbiota

We analyzed the expression of intestinal pro-inflammatory cytokines (e.g. Il-17 and Ifn-γ) and innate immune factors, such as immunoglobulin joining chain (J-chain), polymeric immunoglobulin receptor (pIgR), mucin-2 (Muc2), mucin-4 (Muc4), cryptdins-1 (Crp1), cryptdins-4 (Crp4), cryptdins-1 (Crp5), and lysozyme 2 (Lzy2) in the ileum in this study. The results showed that AKG supplementation increased the mRNA abundance of Crp1, 4, and 5 (*P* < 0.05), but reduced the expression of Muc4 (*P* < 0.05). Interestingly, AKG supplementation significantly inhibited the expression of Crp1 and Crp5 in antibiotic-treated mice (*P* < 0.05). Thus, the influence of AKG in intestinal innate immunity depends on the intestinal microbiota.

## DISCUSSION

AKG plays multiple roles in cellular metabolism, such as regulating amino acid concentration, inhibiting protein catabolism and enhancing protein synthesis [12] and controlling lipid levels [13]. Exogenous AKG can be rapidly transaminated to Glu, Gln, Arg, proline and other amino acids [14, 15]. On the other hand, Glu can be synthesized from Gln or Arg, and Glu is also converted to AKG by glutamate dehydrogenase in the liver, brain or other organs [16, 17]. Our previous reports demonstrated that dietary Arg and Gln influenced growth performance in various conditions of dyshomeostasis, such as mycotoxin contamination, oxidative stress, and infection [18–20], suggesting a similar function for AKG *via* the transamination pathway. In this study, AKG supplementation increased the average feed intake but decreased the daily body weight gain rate. Similarly, oral therapy with AKG lowered the body weight in experimentally induced hypercholesterolemia in rats [21]. The reason might be that AKG reduces fat deposition [22] or improves lipolysis and fatty acid oxidation *via* enhancing Arg and NO synthesis [23]. However, a report has shown that 1% AKG supplementation in the basal diet had little effect on feed intake and weight gain in LPS- challenged piglets [9], while another study has found that 1% AKG increases weight gain, but decreases the feed conversion ratio in the juvenile hybrid sturgeon [24]. The effects of AKG supplementation on body weight and feed intake differ among those investigations. This may be related to the animal models (e.g. mice, rat, pig or juvenile hybrid sturgeon) or the dosage of AKG supplementation.

AKG may affect body weight through changes in gastrointestinal microbial composition. Intestinal microbiota have been suggested to exhibit numerous biological functions and to be involved in the development of various diseases, such as obesity [25], cancer [26], type 2 diabetes [27], metabolic disorders [28], and inflammation [29]. Nutrients play a dominant role in shaping inter-individual variations in intestinal microbial composition [30]. As 95% of exogenous AKG could be metabolized by microorganisms in the gastrointestinal tract [14, 15], AKG may regulate the intestinal microbiota. In this study, we determined that oral AKG influences intestinal microbiota composition, in particular by decreasing the Firmicutes to Bacteroidetes ratio in the feces. The growth of many Bacteroides is restricted by the low pH in the intestinal tract; meanwhile, Firmicutes spp. is more tolerant of acidic pH [31, 32]. Thus, the mechanism of the effect on intestinal microbiota under the regulation of AKG may be that AKG treatment changes the pH in the intestinal tract. Bacteroidetes and Firmicutes are dominant in the human gut, which is consistent with our data that Bacteroidetes and Firmicutes comprise 98.1% and 92.6% of the microbiota in the ileum and feces, respectively. The effect of AKG on lowering body weight may be associated with intestinal microbiota because an increased percentage of Bacteroidetes is positively associated with body weight loss, and the ratio of Firmicutes to Bacteroidetes correlates with obesity [33]. Our previous studies have also shown that Arg or Gln could reduce the Firmicutes to Bacteroidetes ratio in a mouse model [10, 11]. Thus, we speculated that AKG mediates the intestinal microbiota fluctuation, which further influences growth performance in mice.

Gln or Arg has been suggested to play a beneficial role in immunology. Our previous study has shown that Gln improves host defense and decreases specific virulence factor expression in vaccine-immunized mice [34]. Meanwhile, dietary Arg supplementation improves the innate immune response in the mouse intestine [10]. AKG may play a similar role in the intestinal immune response. Previous reports have suggested that AKG is an immune enhancer *via* modulating T cell differentiation and T cell activation [35], managing neutrophil function and generating ROS [5]. AKG activates intestinal innate immunity by improving the expression of α-defensins in the ileum. A-defensins are antimicrobial peptides expressed by Paneth cells or goblet cells, and have important roles in mucosal defense [36]. Interestingly, AKG supplementation inhibits the expression of Crp1 and Crp5 in the ileum of antibiotic-treated mice, suggesting that intestinal microbiota may mediate the influence of AKG supplementation on the expression of α-defensins.

In conclusion, dietary AKG supplementation lowers body weight, influences intestinal microbiota and actives intestinal immunity but fails to increase the body weight and active intestinal immunity in antibiotic-treated mice. It is deduced that AKG lowers body weight and influences intestinal immunity mediated by the change in intestinal microbiota. To our knowledge, this is the first study to systemically investigate the effects of AKG on intestinal microbiota and intestinal immunity. This study aids in enhancing understanding the crosstalk among nutrition, immunity and microbiota.

## MATERIALS AND METHODS

### Animal care

Female ICR (Institute of Cancer Research) mice (aged six weeks) were purchased from SLAC Laboratory Animal Central (Changsha, China). The mice were housed in sterile animal colonies separately (temperature, 25±5°C; relative humidity, 55±5%; 12-h dark/12-h light) and had access to standard rodent feed according to our previous study [10] and drinking water ad libitum. The mice were given 3 days of accommodation before grouping. This study was performed under the guidelines of the Laboratory Animal Ethical Commission of the Chinese Academy of Science. All animal experiments were approved by the Animal Welfare Committee of the Institute of Subtropical Agriculture, Chinese Academy of Sciences (2014-8A).

### AKG supplementation of mice

Sixty mice were randomly separated into two groups (control and AKG, *n* = 30). Mice in the control group received normal drinking water for 2 weeks, while mice in the AKG group received AKG (Sigma-Aldrich Co. LLC, Shanghai, China)-supplemented water (10 g/L). All mice had free access to basal feed in this experiment. The dosage of AKG and the experimental period were selected according to our previous study [12]. Mice were euthanized to collect the luminal contents of the ileum and the feces. All samples were stored at -80 ˚C until further processing. The water intake, feed intake and body weight were recorded during the entire experiment.

### Antibiotic treatment of mice

Sixty mice were randomly assigned into two groups (antibiotics and antibiotics+AKG, *n* = 30). Mice in the antibiotics group received antibiotic-supplemented drinking water (1 g/L ampicillin; 450 mg/L streptomycin; 200 mg/L gentamicin) for 2 weeks according to our previous study [37], while mice in the AKG group received water supplemented with antibiotics and AKG (10 g/L AKG; 1 g/L ampicillin; 450 mg/L streptomycin; 200 mg/L gentamicin). All mice had access to basal feed in the experiment. All mice were euthanized for sample collection, including the ileal luminal contents and the feces. All the samples were stored at -80 ˚C until further processing. The water intake, feed intake and body weight were monitored during the entire experiment.

### 16S rDNA sequencing with Illumina MiSeq Sequencing

The feces and the luminal contents of the ileum were collected for DNA extraction using the Qiagen QIAamp DNA Stool Mini Kit according to the protocol. Equal amounts of DNA from six different mice were pooled to generate one common sample for each type of sample according to our previous study [38]. A commercial biology company (Shanghai Biotree Biotech Co., Ltd., Shanghai, China) performed Illumina MiSeq sequencing and general data analyses.

### Real-time polymerase chain reaction

Total RNA was isolated from liquid nitrogen frozen and ground ileal samples using TRIZOL regent (Invitrogen, USA) according to the manufacturer's recommendations. First strand cDNAs were synthesized using PrimeScript RT reagent Kit with gDNA Eraser (TAKARA BIO INC., Qingdao, China) according to the product manual. Real-time PCR was performed according to our previous study [39]. Primer sequences used in this study were previously reported [40, 41] (Table 1).

**Table 1:** Primer pairs used in the RT-PCR

Gene	ID	Nucleotide sequence of primers (5′–3′)	Product Length
β-actin	NM_007393.3	F: GTCCACCTTCCAGCAGATGT R: GAAAGGGTGTAAAACGCAGC	117
Il-17	NM_010552.3	F: TACCTCAACCGTTCCACGTC R: TTTCCCTCCGCATTGACAC	119
Ifn-γ	NM_008337.4	F: ATGAACGCTACACACTGCATCTTGGCTT R: CCTCAAACTTGGCAATACTCATGAATGC	361
pIgR	NM_011082.3	F: AGTAACCGAGGCCTGTCCTT R: GTCACTCGGCAACTCAGGA	66
J-chain	NM_152839.3	F: GAACTTTGTATACCATTTGTCAGACG R: CTGGGTGGCAGTAACAACCT	88
Muc2	NM_023566.3	F: CCCAGAAGGGACTGTGTATG R: TTGTGTTCGCTCTTGGTCAG	276
Muc4	NM_080457.3	F: GTCTCCCATCACGGTTCAGT R: TGTCATTCCACACTCCCAGA	281
Crp1	NM_010031.2	F: CTAGTCCTACTCTTTGCCCT R: TTGCAGCCTCTTGATCTACA	206
Crp4	NM_010039.1	F: GTCCAGGCTGATCCTATCCA R: GGGGCAGCAGTACAAAAATC	222
Crp5	NM_007851.2	F: GTCCAGGCTGATCCTATCCA R: GATTTCTGCAGGTCCCAAAA	202
Lyz2	NM_013590.4	F: GAATGGAATGGCTGGCTACT R: CGTGCTGAGCTAAACACACC	62

### Statistical analyses

The results were presented as the mean ± the standard error of the mean (SEM). All statistical analyses were performed by IBM SPSS Statistics 22 (IBM Corporation, New York, USA). The statistical analysis between two groups was performed by Student's *t*-test. A *P* value < 0.05 was considered as statistically significant.

## SUPPLEMENTARY FILE



## Abbreviations

AKG: alpha-ketoglutarate
Arg: arginine
Crp1: cryptdins-1
Crp4: cryptdins-4
Crp5: cryptdins-5
Gln: glutamine
Glu: glutamate
Ifn-γ: interferon-gamma
Il-17: interleukin-17
J-chain: immunoglobulin joining chain
LPS: lipopolysaccharide
Lzy2: lysozyme 2
Muc2: mucin-2
Muc4: mucin-4
pIgR: polymeric immunoglobulin receptor
